# Emphasizing the Role of Long Non-Coding RNAs (lncRNA), Circular RNA (circRNA), and Micropeptides (miPs) in Plant Biotic Stress Tolerance

**DOI:** 10.3390/plants12233951

**Published:** 2023-11-23

**Authors:** Anirban Bhar, Amit Roy

**Affiliations:** 1 Post Graduate Department of Botany, Ramakrishna Mission Vivekananda Centenary College, Kolkata 700118, India; 2 Faculty of Forestry and Wood Sciences, Czech University of Life Sciences Prague, 165 00 Prague, Czech Republic

**Keywords:** biotic stress, miRNA, miPEPs, micropeptides, plant–microbe interaction

## Abstract

Biotic stress tolerance in plants is complex as it relies solely on specific innate immune responses from different plant species combating diverse pathogens. Each component of the plant immune system is crucial to comprehend the molecular basis underlying sustainable resistance response. Among many other regulatory components, long non-coding RNAs (lncRNAs) and circular RNAs (circRNAs) have recently emerged as novel regulatory control switches in plant development and stress biology. Besides, miPs, the small peptides (100–150 amino acids long) encoded by some of the non-coding portions of the genome also turned out to be paramount regulators of plant stress. Although some studies have been performed in deciphering the role of miPs in abiotic stress tolerance, their function in regulating biotic stress tolerance is still largely elusive. Hence, the present review focuses on the roles of long non-coding RNAs (lncRNAs) and circular RNAs (circRNAs) in combating biotic stress in plants. The probable role of miPs in plant–microbe interaction is also comprehensively highlighted. This review enhances our current understanding of plant lncRNAs, circRNAs, and miPs in biotic stress tolerance and raises intriguing questions worth following up.

## 1. Introduction

Plant stress response relies primarily on the innate immune system, and upon pathogenic attack, the entire transcriptomic, proteomic, and metabolomic re-programming takes place [1,2]. Intracellular signaling is considered the utmost determinative factor, providing resistance against biotic stress. Small molecules, hormonal cross-talk, and epigenetic regulations contribute immensely to plant immunity [3,4]. MicroRNAs (miRNAs) are another regulatory element controlling vast arrays of plant signaling, including stress response [5]. These miRNAs, along with other non-coding RNAs, e.g., long non-coding RNA (lncRNA), circular RNA (circRNA), etc., were considered part of the non-coding region of the genome and translationally inactive. Recently, it has been reported that many of these non-coding RNAs can translate into functional proteins by utilizing specialized open reading frames (ORFs) [6]. The products of these non-coding RNAs are characteristically different from those of conventional proteins by being significantly smaller in size (≤150 amino acids) and, hence, termed micropeptides (miPs) [7]. The miPs usually lack signal peptides; hence, they are confined within the cytoplasm [8]. However, recently, miPs are also found in different organelles [9,10]. The multifaceted functions of these miPs are now being continuously deciphered and have transformed our understanding of plant stress biology. It is worth mentioning that the study of miPs in plants is in the very preliminary stage and demands more investigation to generate considerable functional data. The role of miPs in abiotic stress tolerance in plants has been studied noticeably, but studies on their role in biotic stress response are scarce. Hence, the present review summarizes the potency of these small peptides in plant biotic stress biology. It comprehensively describes the structural complexity of ORF-producing miPs, the characteristics of principle non-coding RNAs in plants, and their immense capability in combating biotic stress response based on existing knowledge. This review also presents future research perspectives on miPs in combating pathogen attacks in plants.

## 2. Types of Non-Coding RNA (ncRNA) and Their Biological Relevance

Non-coding RNAs are the class of RNA molecules which do not encode any functional protein molecules. This class of RNA molecules includes a diverse group of conventional and non-conventional RNAs. The conventional housekeeping ncRNA, transfer RNA (tRNA), and ribosomal RNA (rRNA) have long been reported and have significant roles in maintaining cellular vitality. Other classes of small non-coding RNAs were discovered during the late 1980s and are continuously being enriched with novel types. The ncRNA pool is responsible for significant cellular functions and constitutes 80% of the total transcriptome mass of a cell [11]. As ncRNAs are heterogeneously originated, they differ in size, function, and biogenesis. Based on their biological functions, ncRNAs are classified into two groups: (i) housekeeping ncRNAs and (ii) regulatory RNAs. Based on size, ncRNAs have been classified according to the 200 nt cut-off, i.e., if they are ≤200 nt in length, they are considered as small ncRNA, and if the size exceeds 200 nt, they are termed as long ncRNA. The housekeeping ncRNA includes tRNA, rRNA, small nuclear RNA (snRNA), and small nucleolar RNA (snoRNA). On the other hand, regulatory ncRNA is further classified into short ncRNA (≤200 nt) and long ncRNA (≥200 nt). The small regulatory ncRNA encompasses microRNA (miRNA), small interfering RNA (siRNA), piwi-interacting RNA (piRNA), trans-activating CRISPR (tracr) RNA, signal recognition particle RNA (7SL), small Cajal body-specific RNA (scaRNA), etc. The long ncRNA includes long intergenic ncRNA (lincRNA), natural antisense transcript (NAT), and circular RNA (circRNA) [12,13]. These ncRNAs control diverse functions, from prokaryotes to eukaryotic higher organisms. The lncRNAs are involved in genome organization, the stability and maintenance of genome size, chromatin structure, compaction, and DNA repair [14]. Recently, it has been observed that many ncRNAs can be translated into small peptides, called micropeptides (miPs). These peptides do not code for any character but regulate many plant and animal functions, including stress response. They are also known to regulate resistance response against biotic and abiotic stress factors. This is an emerging field of study, and many interesting findings have been documented recently. The following sections of this review aim to comprehensively discuss the recent advancement in regulatory roles of two principal ncRNA (lncRNA and circRNA) in biotic stress tolerance in plants and also highlight the enormous possibilities of the regulatory function of miPs in plant immunity, with a special emphasis on miRNA-derived peptides (miPEPs).

## 3. Structural Complexity of Non-Coding RNA

### 3.1. Short Open Reading Frames (sORFs)

**Figure 1:**
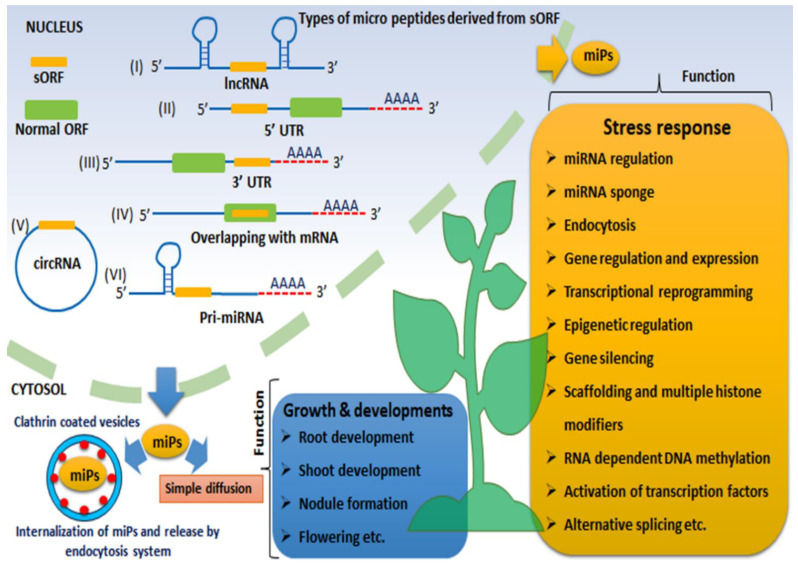
Schematic representation of different types of micropeptides and their roles in plant growth, development, and biotic stress response. Micropeptides originating from sORF are located in (I) long non-coding RNA (lncRNA), (II) 5′ UTR of mRNA, (III) 3′ UTR of mRNA, (IV) sORF over lapping with normal mRNA, (V) circular RNA, and (VI) primary microRNA (pri-miRNA). The miPs are internalized within the cytosol by clathrin-coated vesicles for endocytosis-mediated release or are transported through simple diffusion for cell-to-cell communication during signal transduction.

After the discovery of micropeptides, the repertoire of these fantastic small peptides has continuously been enriched with new members. Their structure, origin, and functional diversity have also regularly expanded (Figure 1). As the most widely accepted terms, peptides having 100–150 amino acids in length are considered as miPs [15]. They are derived from protein-coding or non-coding mRNA, pri-microRNA, or modification from full-length proteins having a secretary or non-secretary nature [16]. Although micropeptides can originate in diverse ways in plants and animals, they have a special open reading frame (ORF) within a genome called a short open reading frame (sORF). The sORFs are usually less than 300 bases in length [17]. This miniscule structure makes them difficult to identify with standard genomics tools. The specialized approach to identifying small transcriptomes by RNA-seq analysis, ribosome profiling (Ribo-Seq), mass spectrometry (ms), and proteogenomics applications can successfully detect miPs and their corresponding sORF [6]. The absence of AUG as a start codon in many micropeptides further obscures the identification procedure. The distinction of sORF from alternative ORF (AltORF) lies in two points: the presence or absence of AUG in sORF but the apparent presence of AUG in AltORF, and the length of the codon in sORF ranging from 10 to 100, but AltORF having a minimum of 30 codons with unrestricted upper limit [18]. This sORF can be found in long non-coding RNAs (lncRNAs), 5′ UTR or 3′ UTR of mRNA, overlapping with mRNAs, circular RNAs (circRNAs), pri-microRNA, and ribosomal RNA [19]. These molecules have diverse functions in plant growth, development, and stress responses (Figure 1). However, the detailed mechanism of micropeptides and non-coding RNAs in controlling the above functions must be deciphered (Table 1).

### 3.2. Long Non-Coding RNAs (lncRNA)

It is more than 25 years since the first lncRNA and XIST (a 17 kb inactive X-specific RNA localized in the nucleus) were reported from animal cells [28]. In plants, early nodulin 40 (ENOD 40) was first identified as lncRNA in *Medicago truncatula* [29]. The lncRNA in plants has a 5′ cap and may be polyadenylated or non-polyadenylated. The non-polyadenylated lncRNA is 50–300 nucleotides long and has a low translational efficiency. Polyadenylated lncRNA mainly originates from intergenic regions and is transcribed by RNA Pol II or RNA Pol V in *Arabidopsis*, *Oryza*, and *Zea mays*. Recently, diverse functions of lncRNA in plants have been identified. RNA-DNA hybrid (R-loop)-associated long non-coding RNAs (lncRNAs), e.g., AUXIN-REGULATED PROMOTER LOOP (APOLO), is found to be involved in DNA methylation and epigenetic control in *Arabidopsis* [30]. A genome-wide analysis of lncRNA in *Camellia sinensis* in response to nitrogen stress revealed a total of 16,452 lncRNAs, out of which 9451 were differentially expressed [31]. The detailed functional variability of lncRNA in plants includes growth and development, flowering and vernalization, light response, seed formation, yield, and stress response [32] (Table 1).

### 3.3. Circular RNAs (circRNA)

Circular RNA is a novel non-coding RNA class originating from pre-mRNA due to a non-canonical splicing process [47]. Usually, splicing gives rise to linear protein-coding mRNA (Figure 1). Sometimes, the covalent attachment of the 5′ and 3′ termini of pre-mRNA produces a closed circular loop of RNA called circRNA. The alternative splicing process of circRNA development is termed “back splicing” [48]. Although circRNA principally consists of exons, they may also contain introns, intergenic regions, 3′ or 5′ UTRs, or they may even be produced from lncRNA [49]. The circRNA do not have any polarity (3′ to 5′ or 5′ to 3′), nor do they possess polyadenylated tails; hence, they are protected from potential ribonuclease attack [50]. For that reason, many viruses use this form of RNA for their propagation. The circularization is usually accelerated due to repetitive and reverse complementary sequences surrounding the splicing sites. The plant circRNA possesses fewer repetitive and reverse complementary regions than that animal circRNA. Plant circRNAs do not potentially act as mi-RNA sponges as they do in animals [51]. Although plant circRNA remains in its infancy, rapid discoveries of novel circRNA classes in plants, genome-wide identification, and their mechanical characteristics open up new dimensions in plant science. Advanced bioinformatic analysis uncovered a large number (95,143) of circRNAs in different plant species (Table 2) [52]. After production and successful circularization, circRNA is primarily retained within the nucleus or transported to the cytoplasm [48]. Besides this nuclear-encoded circRNA in plants, some transposable mitochondrial-encoded circRNAs (mcircRNAs) have also been reported [53]. A separate study revealed that out of 6519 circRNAs in rice, 49.1% are conserved in the *Oryza* genus, and 8.7% showed similarities with entire dicotyledonous plants [54]. This study provides a unique evolutionary relationship of circRNA within plants (Table 1).

### 3.4. MiRNA-Derived Peptides (miPEPs)

MiRNA-derived peptides originate from primary microRNA using single or multiple sORF. The first miPEP was discovered in *Arabidopsis* derived from miRNA171, which targeted the *SCARECROW* gene to control adventitious root formation. Later on, several variants of miRNA171 in different plants controlling similar root development were encountered [55]. The discovery of miPEPs has reformed the gene regulation events in plants, but regulatory mechanisms involving miRNA and miPEPs must be deciphered. The topology of the miRNA gene has shown that sORF is usually present 5′ upstream region. A similar type of organization can be found in different plants, e.g., *Arabidopsis*, *Medicago*, soybean, and grapes, as well as in mammalian cells [16,56,57,58,59]. After the pri-miRNA processes into pre-miRNA by DICER within the nucleus, the remaining portion of pri-miRNA with sORF is transported towards the cytosol. Within the cytosol, they translate into miPEPs, which may act as a transcriptional activator of the miRNA gene where miPEPs function as part of the RNA Pol II transcription complex [34] (Table 1). The external miPEPs may be internalized into the cytosol and transported intracellularly by endocytosis [60] (Figure 1). The miPEPs can virtually be predicted through many bioinformatic tools (Table 2).

**Table 2:** List of databases and bioinformatic tools to decipher plant non-coding RNA and miPs.

Sl. No.	Name of Database	Function	References
1.	PlncRNADB	This is a searchable database of lncRNA sequences and annotation in plants.	[61]
2.	PLNlncRbase	Literature-based database for plant lncRNA for easy curation and determination of biological functions.	[62]
3.	NONCODEV6	Repository of non-coding RNAs in plants and animals. Tissue-specific expression profile of lncRNA.	[63]
4.	PLncDB	Plant lncRNA database. It includes tissues, developmental stages, mutants, stress treatments, and epigenetic regulation of lncRNA.	[64]
5.	Green Non-Coding Database. GREENC	Pipeline to annotate a large number of plant-specific lncRNAs, including algae.	[65]
6.	MiPepid	A Python-based detection software of sORF using FASTA genomic sequences.	[66]
7.	FuncPEP	This database provides functional peptide identification from non-coding portions of the genome.	[67]

## 4. Micropeptides (miPs): Emerging Stars from the “*Dark Matter*” of Biological Sciences

The miPs are challenging to identify and remain concealed within typical RNA structures. The remarkably small size of the protein and specialized open reading frame (ORF) restricted the discovery of miPs in biological sciences for a long time. The rapid advancement of proteo-genomic, transcriptomic, and bioinformatic approaches recently decoded these promising biological regulators. The function of micropeptides was first reported in *Drosophila* [9]. The myoregulin (MLN) was found to be a conserved miP derived from a long non-coding RNA molecule, and regulates muscle contraction in many organisms [9]. Since then, many functional miPs have been reported from different organisms, including plants. For a long time, these crucial regulators derived from the non-coding region of the genome have been obscured from the scientific community; they are considered rising stars from the “dark matter” of biological sciences [68]. Initially, miPs were classified based on size, and small peptides were often overlooked as functionally insignificant. However, recent research has revealed that miPs involve diverse cellular processes, including gene regulation, development, metabolism, and signaling pathways. Although ribosome profiling, mass spectrometry, transcriptomics, and bioinformatic approaches have revealed many miPs in animals and plants, distinguishing miPs from non-functional open reading frames is still difficult. These functional but unannotated ORFs are also called alternative ORFs (altORFs) [18].Different bioinformatic servers and tool kits can decode short open reading frames (sORFs) or probable peptides coded by non-coding RNAs. Some popular and valuable bioinformatic tool kits are tabulated with their functional approaches (Table 2).

## 5. The Regulatory Function of Non-Coding RNA in Biotic Stress Tolerance in Plants

### 5.1. The Role of lncRNA in Biotic Stress Tolerance in Plants

The role of lncRNA in controlling plant pathogenesis is an emerging field, and new studies come up each day with novel mechanistic architecture. The fungal pathogen constitutes a substantial economic loss (USD 100 to USD 200 billion), causing an annual 10–20% crop loss globally (https://www.ars.usda.gov, accessed on 18 November 2023). In *Arabidopsis*, several lncRNAs were found to be induced after infection of *Fusarium oxysporum*. Among 159 long non-coding transcriptionally active regions (lncTARs), 20 were found to be *Fusarium*-specific [69]. Later, many lncRNAs were reported from *Arabidopsis* in response to the translation elongation factor Tu (elf18). These lncRNAs were known to induce plant immune response against *Pseudomonas syringe* pv tomato DC3000 [70]. Despite the biotic stress response in plants, another exciting finding has recently surfaced in the case of insect pest-plant–pathogen interaction. *Plutella xylostella* is a notorious pest for cruciferous crops and exhibited lncRNA-mediated regulatory networks during infection with *Metarhizium anisopliae* [71]. The RNA-Seq analysis of sunflowers in response to *Sclerotinia* head rot disease revealed a high accumulation of lnc-RNA and resistance response in redox homeostasis and cell wall reinforcement [72]. The lncRNAs were found to be induced in *Vitis vinifera* (grapevine) in response to the gray mold fungus *Botrytis cinerea*, where they controlled chitin degradation, glutathione metabolism, and stilbenoid biosynthesis [73]. A novel lncRNA, MuLnc1, was reported from Mulberry in a similar type of stress response (Gai et al., 2018 [21]). Recently, a genome-wide analysis in rice against *Magnaporthe oryzae* has identified ≥2600 lnc-RNAs [74]. Similarly, many intergenic lncRNA candidates were also identified in rice against *Magnaporthe oryzae* (Jain et al., 2017 [28]). In melon (*Cucumis melo* L.), a total of 539 lncRNAs were reported in response to powdery mildew pathogen in both powdery mildew-resistant (MR-1) and susceptible melon (Top Mark). The differential expression patterns have confirmed 254 lncRNAs to be mildew-specific, while 42 were found to control mi-RNA expression and network [75]. Many lncRNAs were reported from plant-*Phytophthora* infection, i.e., as discussed earlier, the long non-coding RNA (lncRNA) of *FL7* (*nalncFL7*) is highly expressed in *Arabidopsis* against *Phytophthora capsici* (Ai et al., 2023 [23]). Similarly, lncRNA33732 was a positive regulator in resistance response against *P. infestans* (Cui et al., 2019 [24]). Genome-wide identification studies have identified 2857 lncRNAs against *P. infestans* in potatoes (Cao et al., 2021 [32]), and 2363 lncRNAs were reported from *C. pepo* in response to *Phytophthora xanthii* infection (Tian et al., 2022 [31]). Different lncRNAs were reported from cotton in response to *Verticillium dahliae* (Zhang et al., 2018; Li et al., 2022 [26,29]). In wheat, lncRNAs were characterized and identified against varied pathogens, i.e., about 125 lncRNAs were identified in response to *Blumeria graminis* f. sp. *tritici* and 1319 long intergenic non-coding RNAs (lincRNAs) were identified against *Rhizoctonia cerealis* (Xin et al., 2011; Yi et al., 2023 [25,27]) (Table 1).

Among bacterial diseases, *Citrus* infected with *Candidatus Liberibacter asiaticus* (CLas) bacteria showed a massive induction of lncRNA [76]. The beneficial rhizobacteria *Bacillus subtilis* SL18r can cause a systemic induced resistance response (ISR) in tomato plants against the foliar pathogen *Botrytis cinerea*. This interaction evidenced the instigation of lncRNA MSTRG18363 by employing the decoy system miR1918 [77]. Interestingly, the rhizosphere bacteria *Pseudomonas putida* Sneb821 counteracts *Meloidogyne incognita* by inducing ncRNA44664 in tomatoes [78]. The lncRNA induced after a pathogen attack may interact with the hormonal signaling pathway in plants. In rice lncRNA, *ALEX 1* was co-expressed with the jasmonic acid (J.A.) signaling pathway in response to *Xanthomonas oryzae* pv. *oryzae* (Xoo) [79]. On the other hand, a conjoint genome-wide analysis of lncRNA and the expression of genes in poplar (*Populus* × *euramericana*) during exogenous salicylic acid (S.A.) treatment revealed lncRNA–mRNA interactions and an S.A.-mediated defense response [22].

### 5.2. The Roles of circRNA in Biotic Stress Tolerance Circuitry in Plants

The roles of circRNA in abiotic stress tolerance have recently been extensively studied. The genome-wide analysis, RNA-Seq, and other bioinformatic analyses have revealed vast arrays of circRNAs in response to salinity, drought, heat, U.V., chilling, heavy metal stress, etc. [41,80,81,82,83]. The same for the biotic stress tolerance in plants is still elusive. The expression analysis of the circRNAs of *Arabidopsis thaliana* in response to fungal and bacterial pathogens has revealed that the exonic circRNAs are intriguingly involved in immune response in both cases. The circR194 and circR4022 were reportedly involved in *Pseudomonas syringae* infection, whereas circR11208 showed a resistance response against *Botrytis cinerea* infection [40]. The comparative transcriptomic analysis of melon against powdery mildew disease unveiled considerable numbers of circRNAs differentially expressed after infection and reported significantly modulating miRNA interaction [84]. Maize Iranian mosaic virus (MIMV) infects maize and some other members of Poaceae. MIMV has activated many miRNAs in maize, and bioinformatic analysis has revealed numerous target sites of circRNA within those miRNAs [85], endorsing the circRNA–miRNA interaction within plants in response to biotic stress. Among many non-coding RNAs, circRNA was also evident in different stress responses in leguminous plants. Still, their detailed roles in biotic stress tolerance are limited [39]. Similarly, kiwi fruit infected with the *Pseudomonas syringae* expressed 584 circRNAs in response to pathogenesis. The involvement of circRNA in biotic stress is evident and explored with more plant–microbe interaction studies. The involvement of circRNA in both biotic and abiotic stress has also been documented in tomato [33]. Studies of the in-depth mechanistic roles, interaction pattern, and mode of action with other immunogenic modules are urgently necessary to unravel the mystery behind this repertoire of non-coding molecules. Recently, it has been reported that circRNA may control transcriptomic reprogramming by transposon-mediated gene expression during stress response in plants [41,86]. Some other interesting roles of circRNAs in biotic stress tolerance are documented in Table 1.

### 5.3. The miPEPs and Biotic Stress Tolerance in Plants

There is limited information on the specific role of miPEPs in biotic stress tolerance in plants, but active research in this area continues to unravel the most exciting findings. The miRNAs are known to regulate gene expression at the post-transcriptional level, controlling various biological processes, including stress response. Recently, it has been reported that miPEPs may regulate the expression of different defense-responsive genes [36]. The expression of target genes is modulated by miPEPs in different immunomodulatory actions [36]. Diverse mi-RNAs are reported to control the hormonal cross-talk in response to biotic stress tolerance in plants [87]. Hence, the miPEPs might also modulate the expression of this hormone-signaling pathway. Salicylic acid (S.A.), jasmonic acid (J.A.), and ethylene (E.T.) are known to play essential roles in biotic stress responses, and the interaction between miPEPs and these hormonal pathways could influence stress tolerance in plants. Recently, it has been observed that more than 7000 small protein-coding genes have existed in the *Arabidopsis* genome that may produce small hormone-like peptides controlling long-distance interorgan or cell-to-cell signaling in plants [88]. The mechanistic role of miPEPs in controlling such hormone-like peptides may also open up novel dimensions of plant stress biology. MiPEPs may also be involved in epigenetic regulation in plants as miRNA is well characterized as a modulator of epigenetic control. The miR166, miR168, miR393, miR397, miR398, miR1524, and miR2119 were recently reported to be involved in epigenetic regulation and the development of the heart stage in *Coffea canephora* during embryogenesis [89]. The different targets of miPEPs in regulating miRNA have been studied in *Medicago truncatula* and *Arabidopsis thaliana* (de Bruijn et al., 2020 [38]). Similar targets were also investigated in *Arachis hypogaea* (Ram et al., 2019 [37]) (Table 1).

## 6. The Modes of Action of Non-Coding RNAs in Plant Immune Response

### 6.1. Plant Immunity

The plant immune system is coordinated by an array of receptors, both extracellular and intracellular, for the smooth dissipation of signals. Usually, cell surface receptors (pattern-triggered receptors, PRR) interact with the cognate pathogen-specific molecules (microbe-associated molecular pattern, MAMP, or pathogen-associated molecular pattern, PAMP). This interaction is the first and most crucial step of pathogen recognition (recognition reaction) and is responsible for the first line of defense response called PAMP-triggered immunity (PTI). The second line of defense is instigated against specific toxins released by the pathogen, i.e., effectors. The plants exhibit a more robust and high amplitude defense response against the pathogen, called effector-triggered immunity (ETI) [90]. Subsequently, several downstream signaling cascades are successively activated and ultimately regulate the expression of defense-responsive genes [1]. Reactive oxygen species (ROS) and calcium ions are the most common secondary messengers activated in due course [1,91,92]. The non-coding RNAs have the paramount scope to interact with any downstream defense signaling.

### 6.2. Immune Sensing and Signaling

**Figure 2:**
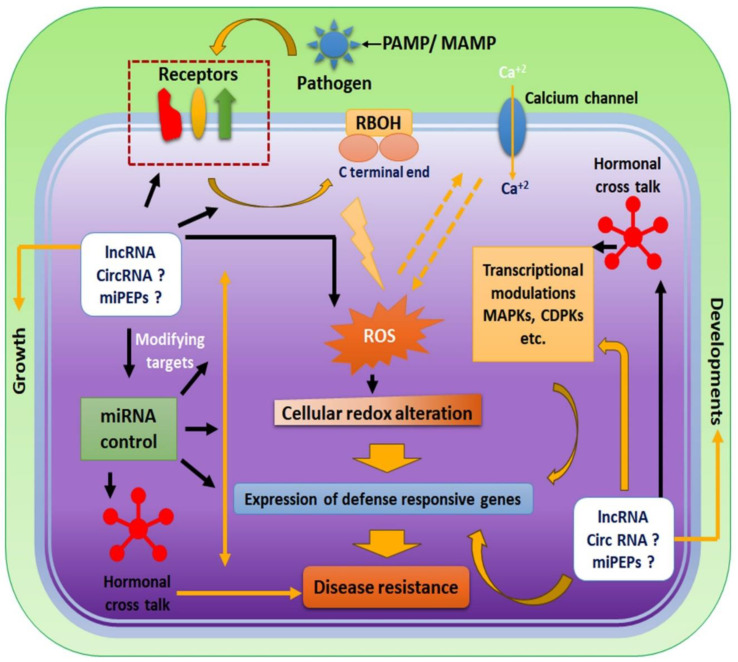
The immune control mechanism in plants by miPs (lncRNA, circRNA, and miPEPs). Receptors on the host surface detect the PAMPs or MAMPs associated with the pathogen. The successful recognition reaction leads to respiratory burst oxidase (RBOH) activation, leading to cellular redox alteration and subsequent calcium channel activation. ROS and calcium influx perpetuate a mutual activation cycle. The redox alteration and calcium influx readily activate transcription modulators, e.g., MAP kinases (MAPKs) and calcium-dependent protein kinases (CDPKs). The consorted internal signaling leads to disease resistance by varied defense-responsive gene expression. The microRNAs (miRNAs) have an intricate relationship with defense response in plants. There are many miRNA targets in the plant defense response pathway, which can modulate resistance response in multitier control modules. The lncRNA, circRNA, and miPEPs can alter or mimic the targets of these miRNAs and, hence, have multifaceted control over plant immunity. Some studies report the direct correlation of lncRNA with miRNA and mRNA networks in plant defense response. The regulatory action of other forms of miPs is urgently required to decipher the complete scenario of the control mechanism of these small regulators in plant immunity. These non-coding RNAs also control the balance between healthy and diseased conditions in plants.

The PAMP elongation factor-Tu (EF-Tu) dramatically induces lncRNA At5NC056820 in *Arabidopsis* [93]. Similarly, flg22 from *Pseudomonas fluorescens* causes the prominent induction of lncRNAs in tomatoes [94]. The indirect modulation of immune-responsive proteins is also documented in plants. The miR482 has targeted the coiled-coil domain of the N terminal region of NLR (nucleotide-binding site–leucine-rich repeats) genes in *Solanum* sp. [95]. The tomato lncRNA23468 mimics the target of the above miR482, inhibiting the repression of NLR and enhancing tomato resistance to *Phytophthora infestans* [96]. The lncRNA may act as a decoy in controlling plant immunity. In an independent study, tomato lncRNA15492 and lncRNA08489 have resulted in the over-expression of NLRs by decreasing miR482a and miR482e-3p [97,98]. In a recent study, it has been reported that in photophilic rice plants, miR172 helps in juvenile to flowering stage conversion (Dash et al., 2023 [99]). Genome-wide analysis and endogenous RNA (ceRNA) network studies revealed that some lncRNAs potentially targeted miR172 in tomatoes during *Phytophthora infestans* infection (Cui et al., 2019 [24]). Some miRNAs from wild and crop varieties of rice, e.g., miR397, miR407, and miR168, are capable of sensing biotic and abiotic stress combinations through the involvement of lncRNA coregulatory targets (Biswas et al., 2021 [20]; Dash et al., 2022 [100]). The immune sensing and regulation of quantitative trait loci (QTL) controlling multiple stress factors is an emerging field. The discovery of lncRNA targeting QTL, affecting both biotic and abiotic stress, will revolutionize agronomy and could minimize the global food crisis (Mahapatra et al., 2023 [101]). Some lncRNAs are reported to control calcium signaling downstream, e.g., MuLnc1 in Mulberry acts as a downstream signaling modulator in the calmodulin pathway and ROS production. The lncRNA *salicylic acid biogenesis controller 1* (*SABC1*) is known to control the balance between healthy and diseased plants. *SABC1* recruits the polycomb repressive complex 2 to suppress the NAC domain-containing transcription factor 3 (*NAC3*). *NAC3* is responsible for the activation of *isochorismate synthase 1* (*ICS1*), a key enzyme catalyzing salicylic acid (S.A.) [102]. Recently, the antisense lncRNA of *FL7* (*nalncFL7*) was reported to activate the MAPK signaling cascade to impart resistance response [23]. Non-coding RNAs control many developmental and physiology-related transcriptional modules. There are some well-characterized lncRNAs, e.g., *COOLAIR*, *COLDAIR*, and *COLDRAP*, which are known to modulate Flowering locus *C* in *Arabidopsis* [103], and *TWISTED LEAF* reported in rice to control R2R3-MYB [104]. The *Arabidopsis* lncRNA ELF18-INDUCED LONG-NONCODING RNA1 (ELENA1) imparts significant resistance against Pst DC3000, which, with the help of a mediator molecule (MED36a), induces pathogenesis-related 1 (*PR1*) gene expression [70,105]. There are many lncRNAs, e.g., LncRNA42705/lncRNA08711, lncRNA39896, and lncRNA11265/lncRNA15816, which are known to modulate the target of miRNAs (miR159, miR166b, and miR164a-5p) where they function as a decoy in plant immunity [106] (Figure 2).

### 6.3. Reactive Oxygen Species (ROS) and Hormonal Cross-Talk: A Key Player in Non-Coding-RNA-Mediated Defense Signaling in Plants

ROS is a customary plant reaction against diverse pathogens, i.e., bacteria, viruses, fungi, nematodes, or even eukaryotic pathogens [91,107]. After interaction with the pathogen, the redox alteration is an inherent part of plant immunity [1]. The exclusive root-invading pathogen can sometimes accumulate ROS in shoot tissue [108]. The degree of accumulation and balance of ROS varies with the progression of the disease. Both the PTI and ETI of the plant immune cycle can accumulate ROS, and channel them to hypersensitive response (H.R.) or downstream signaling [109]. A comparative transcriptomic study in tomatoes against *Phytophthora infestans* has revealed the regulatory mechanism of lncRNA in cellular redox homeostasis. The lncRNA16397 was reported to regulate the function of glutaredoxin to alleviate oxidative damage in the cell [110]. The intricate interaction of lncRNA with the ROS pathway is widely studied in human pathogenesis, e.g., ROS balance is mediated by *Wolbachia* in *Aedes aegypti*-mediated dengue fever [111]. Hence, oxidative balance in the cellular milieu is a universal phenomenon. In some abiotic stress responses in plants, the interconnection of redox status and stress reactions was also established; e.g., lncRNA973 was reported to control salt stress in cotton by modulating the ROS pathway [112]. The detailed lncRNA mining in *Vitis vinifera* infected with obligate biotrophic fungus *Erysiphe necator* (powdery mildew disease) and *Plasmopara viticola* (downy mildew disease) has revealed the direct interaction of lncRNAs with the redox signaling pathway in the resistance response of grapevine [113]. In the rice plant, an mRNA-lncRNA network was constructed in response to Rice Black-Streaked Dwarf Virus infection, and at least 20 differential lncRNA were reported. They may directly correlate with cellular calcium accumulation and ROS production [114]. On the contrary, *Citrus tristeza* virus (CTV) was known to develop a lncRNA called low-molecular-weight tristeza 1 (LMT1), which can modulate host redox status during pathogenesis [115]. *Arabidopsis thaliana* BPA1-LIKE PROTEIN3 (BPL3) is an RNA-binding protein known to suppress ROS production during pathogenesis. In a separate study, it was observed that BPL3 suppresses FORKED-LIKE7 (FL7) transcript accumulation by synthesizing the long non-coding RNA (lncRNA) of FL7 (nalncFL7) [23]. Although direct interaction is unknown, circRNA competitively binds the miRNA target and regulates plant immune response in different plants [116].

The production of ROS and subsequent resistance response in plants are also connected to the hormonal cross-talk. ABA and ethylene have been known to control redox homeostasis in the intracellular milieu [117]. The ROS wave is also connected through calcium signaling, hydraulic waves, and electrical signals. This signaling concave has been coordinated mainly by salicylic acid and jasmonic acid in an antagonistic way [118,119,120]. The role of lncRNA, circRNA, and miPEPs in coordinating the hormonal response is well documented in plant growth, development, and abiotic stress response [121,122]. The investigation concerns the roles of different non-coding RNAs in regulating biotic stress response by modulating the expression of different genes, inhibiting expression, altering binding preferences, modifying miRNA targets, RNA-DNA hetero duplex formation, etc., radically revolutionizing the plant immune biology [123]. More research is needed to interpolate the non-coding RNA with hormonal cross-talk and other established resistance pathways within plants [124,125] (Figure 2).

## 7. Conclusions and Future Directions

Although micropeptide (miP) research is in its early stage in biological sciences, considerable developments have been observed in animal science, particularly concerning disease and therapeutics. In plants, many micropeptides derived from non-coding regions of the genomes have now been deciphered, and there will be many more to come in the future. The preliminary data suggest that these excellent regulatory small peptides could now be considered the tip of the iceberg. Nevertheless, miPs are emerging as imperative players in the complex network of responses that plants engage in to withstand biotic stress. Understanding the functions of these small peptides could have implications for formulating strategies to enhance crop resilience and improve agricultural productivity in the face of challenges posed by pathogens and other biotic stressors. More focused studies considering plant miPs will provide more insightful findings and mechanistic evidence concerning the plant–pathogen interaction.

The following future research directions may be capable of putting the missing pieces together:

1. The complete micropeptide map of major crop plants and model plants is necessary.
2. A stringent yet feasible toolkit and advancing the presently available bioinformatic platform are required.
3. An in-depth study is required to decipher the precise functions of miPs in plant–microbe interaction.
4. The updating of the existing plant immune system with the proper incorporation of miPs in pathogen recognition, interaction with pattern-triggered immunity (PTI), effector-triggered immunity (ETI), and intracellular signaling is also required.
5. Plant immunity is multifaceted signaling. Hence, the interaction of stress-associated miPs with other signaling intermediates and hormonal cross-talk will provide more insights.
6. The interaction networks between miP and miRNA need more dissection and follow-ups.
7. The potential targets of miPs for genetic engineering and genome editing tools, e.g., clustered regularly interspaced short palindromic repeats (CRISPR) targets of miPs, may revolutionize plant science research in the Anthropocene.
8. Dedicated studies on the diversity, conservation, and evolution of miPs among plant species may unveil the eco-evolutionary aspects of plant–pathogen interactions.

## Figures and Tables

**Table 1:** List of lncRNA, circRNA, and miPEPs involving plant biotic stress responses.

Sl. No	Name of Plant	Name of Pathogen	Interaction	References
lncRNA				
1.	Plant	Biotic stress	miRNA–lncRNA interaction	[20]
2.	Mulberry (*Morus multicaulis*)	*Botrytis cinerea* and *Pseudomonas syringae* pv tomato DC3000	MuLnc1-driven inactivation of calmodulin-like protein gene CML27	[21]
3.	Cotton (*Gossypium hirsutum* L.)	Aphid	GhlncRNA149.1 interacts with the CC-NBS-LRR family gene GhA01G0129 as a potential target.	[22]
4.	*Arabidopsis thaliana*	*Phytophthora capsici*	nalncFL7 negatively regulates *FORKED-LIKE7* (*FL7*).	[23]
5.	Tomato (*Solanum lycopersicum* L.)	*Phytophthora infestans*	lncRNA33732 interaction with *WRKY1*	[24]
6.	Wheat (*Triticum aestivum*)	*Blumeria graminis* f. sp. *tritici*	Non-coding RNA profiling	[25]
7.	Cotton (*Gossypium hirsutum* L.)	*Verticillium dahliae* and *Botrytis cinerea*	GhlncNAT-*ANX2*- and GhlncNAT-RLP7 control the expression of *LOX1* and *LOX2*.	[26]
8.	Wheat (*Triticum aestivum*)	*Rhizoctonia cerealis*	MSTRG.4380.1 in growth retardation of fungi	[27]
9.	Rice (*Oryza sativa*)	*Magnaporthae oryzae*	Intergenic lncRNA candidates for resistance	[28]
10.	Cotton (*Gossypium hirsutum* L.)	*Verticillium dahliae*	Overexpression of lncRNA012077 and down regulation of lncRNA007722	[29]
11.	Rice (*Oryza sativa*)	*Ustilaginoidea virens*	UvlncNAT-MFS, development of smut fungus	[30]
12.	Pumpkin (*Cucurbita pepo* L.)	*Phytophthora xanthii*	lncRNA modulates immune responsive pathway, MAPK pathway, and hormonal cross-talk.	[31]
13.	Potato (*Solanum tuberosum* L.)	*Phytophthora infestans*	Genome-wide analysis of lncRNA and their interrelationship	[32]
14.	Tomato (*Solanum lycopersicum* L.)		miRNA–lncRNA interaction, biotic/abiotic stress tolerance	[33]
miPEPs				
15.	Plants (*Arabidopsis*, grapevine, soybean, and *Medicago*)	-	Interaction network of miPEPs as transcription factor, endocytosis, and transcriptional activator	[34]
16.	Plants	-	Discovery of miPEPs in their probable role in plants and animals	[35]
17.	Plants	-	miPEPs in growth, development, and stress response	[36]
18.	Peanuts (*Arachis hypogaea*)	-	Mining of miRNA and their potential targets of miPEPs	[37]
19.	*Medicago truncatula* and *Arabidopsis thaliana*	-	Finding the role of miPEPs in regulating the expression of miRNA and development of tasi-RNA and phasi-RNA	[38]
CircRNA				
20.	Legume crops	-	Interaction with DNA, RNA, and protein, modulation of target protein	[39]
21.	*Arabidopsis thaliana*	*Pseudomonas syringae* and *Botrytis cinerea*	circR194 and circR4022 involved in resistance to *P. syringae*, and circR11208 protecting from *B. cinerea*	[40]
22.	Plants	-	Bioinformatic mining of circRNA and their potential roles in biotic and abiotic stress factors	[41]
23.	Plants	-	Identification of circRNA in plants with reference to biotic/abiotic stress	[42]
24.	Plants	-	Post-transcriptional modification of gene expression	[43]
25.	Tea plant (*Camellia sinensis*)	*Helopeltis theivora*	Activation of secondary metabolites, endogenous target mimics (eTMs) of target genes, e.g., aspartyl protease, phospholipase, lectin receptor, etc.	[44]
26.	Tomato (*Solanum lycopersicum* L.)	Planticine^®^-induced defense responses	Upregulation of circRNA whitefly-induced gp91	[45]
27.	Tomato (*Solanum lycopersicum* L.)	*Phytophthora infestans*	circRNA45 and circRNA47, positive regulators of resistance response in tomato	[46]
